# One-Step High-Temperature-Synthesized Single-Atom Platinum Catalyst for Efficient Selective Hydrogenation

**DOI:** 10.34133/2020/9140841

**Published:** 2020-04-29

**Authors:** Qingyuan Bi, Xiaotao Yuan, Yue Lu, Dong Wang, Jian Huang, Rui Si, Manling Sui, Fuqiang Huang

**Affiliations:** ^1^State Key Laboratory of High Performance Ceramics and Superfine Microstructure, Shanghai Institute of Ceramics, Chinese Academy of Sciences, Shanghai 200050, China; ^2^State Key Laboratory of Rare Earth Materials Chemistry and Applications, College of Chemistry and Molecular Engineering, Peking University, Beijing 100871, China; ^3^Institute of Microstructure and Properties of Advanced Materials, Beijing University of Technology, Beijing 100124, China; ^4^Shanghai Synchrotron Radiation Facility, Shanghai Institute of Applied Physics, Chinese Academy of Sciences, Shanghai 201800, China

## Abstract

Although single-atom catalysts significantly improve the atom utilization efficiency, the multistep preparation procedures are complicated and difficult to control. Herein, we demonstrate that one-step *in situ* synthesis of the single-atom Pt anchored in single-crystal MoC (Pt_1_/MoC) by using facile and controllable arc-discharge strategy under extreme conditions. The high temperature (up to 4000°C) provides the sufficient energy for atom dispersion and overall stability by forming thermodynamically favourable metal-support interactions. The high-temperature-stabilized Pt_1_/MoC exhibits outstanding performance and excellent thermal stability as durable catalyst for selective quinoline hydrogenation. The initial turnover frequency of 3710 h^−1^ is greater than those of previously reported samples by an order of magnitude under 2 MPa H_2_ at 100°C. The catalyst also shows broad scope activity toward hydrogenation containing unsaturated groups of C=C, C=N, and C=O. The facile, one-step, and fast arc-discharge method provides an effective avenue for single-atom catalyst fabrication that is conventionally challenging.

## 1. Introduction

Single-atom catalysts (SACs) with unique electronic/geometric structures exhibit more effective atom utilization and can act as a promising bridge between homogeneous and heterogeneous catalysis [1–14]. The stability of SACs is a critical issue for their practical applications [15–17]. It was reported that the presence of subnanometer clusters in SACs can affect the overall thermal stability [18–23]. Tremendous efforts are made to improve the thermal stability by enhancing the metal-support interactions [1–17]. Currently, most SACs are prepared by the confinement and coordination of active metal atoms to the defective substrates via adsorption [24, 25], coprecipitation [1, 26], atomic layer deposition [27], galvanic replacement [28–30], and ionic exchange [11, 31] under mild conditions. However, the multistep procedures and complicated processes generally breed the inevitable difficulty in precise controlling of the atomic dispersion. It can also lead to the waste of resources and the generation of environmentally unfriendly by-products. Most recently, high-temperature-assisted routes (e.g., pyrolysis, fusion, and high-temperature shockwave) have been successfully utilized to synthesize the SACs and attract ever-increasing attention [32–36]. It was widely accepted that high temperature can significantly facilitate the formation of metal-support bonds or enhance the metal-atom-support interactions and thus result in excellent thermal stability [32–36]. Nevertheless, high-temperature (>3000°C) synthesis of SACs remains a challenge.

Herein, we present the use of high-temperature arc discharge to directly synthesize and stabilize platinum (Pt) single atoms in molybdenum carbide (MoC) substrate at ultrahigh temperatures (up to 4000°C). Compared to the traditional metal oxides, MoC shows excellent electrical conductivity which is beneficial for the electron transfer between active Pt sites and substrate and the formation of unique electronic/geometric structures. In contrast to the previously reported results [37], our MoC substrate is a typical single crystal with a small particle size. The high-temperature arc-discharge process provides sufficient activation energy for the formation of MoC substrate and strong Pt-MoC interactions, which is critical to achieve the Pt single-atom dispersion for overall stability in practical applications. This facile one-step synthesis strategy under extreme conditions can avoid the uncontrollable factors generally existing in conventional multistep procedures and largely shorten the preparation time to only tens of minutes. The high-temperature-stabilized single-atom Pt catalyst (Pt_1_/MoC) shows outstanding performance and excellent stability for the selective hydrogenation reaction, which is a key process for the synthesis of drugs, dyes, agrochemicals, and many biologically active intermediates [38–44]. Importantly, Pt_1_/MoC exhibits a turnover frequency (TOF) greater than those of existing catalysts by an order of magnitude in quinoline hydrogenation under mild conditions. Furthermore, the versatile Pt SAC shows a broad scope activity toward selective hydrogenation containing unsaturated groups of C=C, C=N, and C=O.

## 2. Results and Discussion

**Figure 1:**
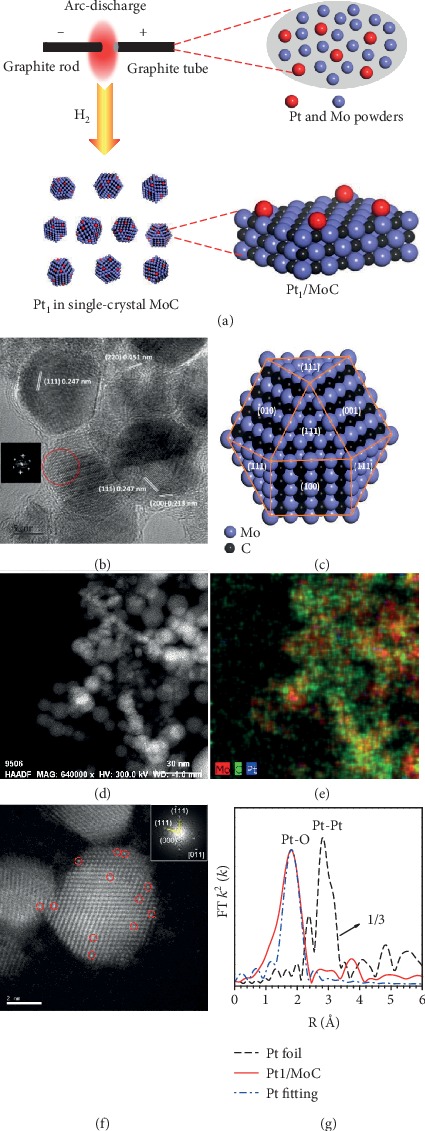
(a) One-step high-temperature synthesis of Pt_1_/MoC catalyst via arc-discharge route. (b) HRTEM image and (c) crystal structure of single-crystal MoC. The crystal plane parameters and selected fast Fourier transform (FFT) results are demonstrated in (b). (d) HAADF-STEM image; (e) EDS Mo, C, and Pt mappings; and (f) aberration-corrected STEM-HAADF image of Pt_1_/MoC. The presence of atomically dispersed Pt in (f) is highlighted by the red circles. Inset in (f) is the FFT data of Pt_1_/MoC. (g) FT-EXAFS spectra of Pt_1_/MoC and bulk Pt foil at the Pt *L*_3_-edge, showing the surrounding atoms adjacent to Pt atoms.

MoC and Pt/MoC were synthesized via the facile one-step high-temperature arc-discharge strategy (Figures 1(a) and S1) [45, 46]. Note that the arc discharge process only takes tens of minutes (Figure S2), which can largely shorten the overall preparation time. As depicted in Figures 1(b) and 1(c), the pure single-crystal MoC with a considerable specific surface area (Figure S3 and Table S1) was readily obtained. The Pt nanoparticles (NPs) and even Pt single atom in MoC were also *in situ* formed, as shown in Figures 1(d)–1(g). X-ray diffraction (XRD) patterns presented in Figure S4 clearly show the exclusive presence of MoC phase with the face-centered-cubic *α*-MoC structure [37]. The absence of any Pt-containing phases indicates the high dispersion of Pt species. High-resolution transmission electron microscopy (HRTEM) data of 1% Pt/MoC (Figure S5) demonstrates the formation of small Pt NPs with a diameter of 2.2 ± 0.2 nm. Clearly, the particle size decreases with the Pt loading decreasing. No appearance of Pt NPs was observed in Pt_1_/MoC. High-angle annular dark field- (HAADF-) scanning transmission electron microscopy (STEM) combined with energy-dispersive spectroscopy (EDS) reveals the formation of atomic Pt in Pt_1_/MoC (Figures 1(d), 1(e), and S6). Aberration-corrected STEM-HAADF images exhibit direct observation of the Pt single atoms (Figures 1(f) and S7). Pt dopant gave no change on the single crystal nature of matrix (inset in Figure 1(f)), and the isolated Pt atoms were tenaciously anchored in MoC substrate. To further verify the atomically dispersed Pt in Pt_1_/MoC, Fourier transform-extended X-ray absorption fine structure (FT-EXAFS) spectra were performed (Figures 1(g), S8, and S9 and Table S2). Only one notable peak at ca. 2 Å from the Pt-O contribution is observed, and no signal in the region 2.5 to 3 Å from the Pt-Pt contribution appears, indicative of the sole presence of single-atom Pt in Pt_1_/MoC (Table S2) [1, 37].

**Figure 2:**
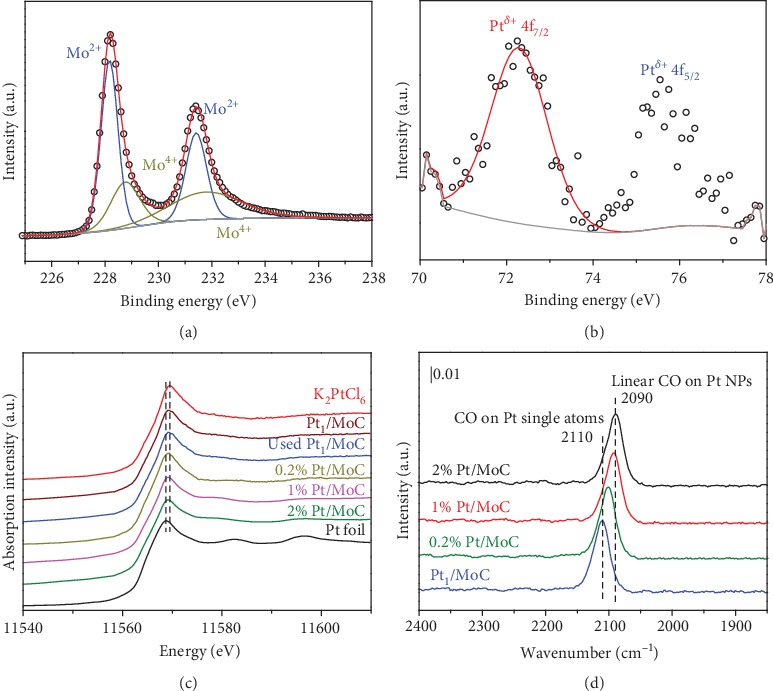
XPS (a) Mo 3d and (b) Pt 4f of Pt_1_/MoC. (c) Pt *L*_3_-edge XANES and (d) *in situ* DRIFTS of CO adsorbed on Pt-based samples.

X-ray photoelectron spectroscopy (XPS) data of Mo 3d for Pt_1_/MoC catalyst demonstrates that Mo exists in Mo^4+^ and Mo^2+^ states (Figure 2(a)). Pt 4f XPS depicted in Figures 2(b) and S10 show that the atomically dispersed Pt is in the Pt^*δ*+^ state, indicative of the unique electron structure of Pt single atoms in Pt_1_/MoC [1, 37]. Normalized X-ray absorption near-edge structure (XANES) spectra at the Pt *L*-edge of Pt/MoC show a visible blue-shift from Pt foil to Pt_1_/MoC (Figure 2(c)), indicating that the Pt single atoms in Pt_1_/MoC possess positive charges in ambient atmosphere. *In situ* diffuse reflectance infrared Fourier transform spectroscopy (DRIFTS) was conducted to detect the CO chemical adsorption on Pt/MoC (Figure 2(d)). The band at 2090 cm^−1^ is ascribed to CO linearly adsorbed on Pt NPs in 2% Pt/MoC. As Pt size decreases, significant blueshift occurs for CO adsorption (2093 and 2101 cm^−1^ for 1% Pt/MoC and 0.2% Pt/MoC, respectively). The band at 2110 cm^−1^ is assigned to CO adsorbed on atomically dispersed Pt species, further confirming the fine isolation of Pt atoms in Pt_1_/MoC catalyst [1–3, 37].

Quinoline hydrogenation was selected as a model reaction to evaluate the catalytic activity of Pt/MoC. All the Pt/MoC catalysts were found to be highly efficient for selective quinoline hydrogenation under 2 MPa H_2_ at 100°C, as the selectivity of >99% to 1,2,3,4-tetrahydroquinoline listed in Table 1. No trace of frequently generated by-products like 5,6,7,8-tetrahydroquinoline or decahydroquinoline was observed. As Pt size decreases, the average TOF significantly increases from 393 h^−1^ for 2% Pt/MoC to 766 h^−1^ for 1% Pt/MoC to 1921 h^−1^ for 0.2% Pt/MoC (Table 1, entries 1-4). Remarkably, Pt_1_/MoC shows outstanding average TOF of 3380 h^−1^ (initial TOF of 3710 h^−1^) and near full conversion (>99%) for quinoline transformation (entries 5 and 6). Noteworthy, the exceptional TOF is greater than those of previously reported catalysts by an order of magnitude for quinoline hydrogenation under mild conditions (Table S3) [42–44, 47–49]. Pt_1_/MoC can also achieve selective reaction at low temperature of 60°C (entry 7) or under low H_2_ pressure of 1 MPa (entry 8). For the Pt catalysts with similar loading and comparable particle size (Figure S11) tested, the use of MoC rather than NbC, WC, and TiC as the most suitable support can significantly stimulate the catalysis potential of Pt (entries 9-11). In contrast, Pd, Ru, and Au with an identical size on MoC (Figure S11) show low efficiency for 1,2,3,4-tetrahydroquinoline synthesis. Only the catalysts based on Pt could deliver the high activity and provide the intrinsic advantages (entries 12-14). Compared to the one-step high-temperature strategy, Pt/MoC prepared by the conventional impregnation method (Figure S12) displays lower activity with the average TOF of 659 h^−1^ (entry 15). The commonly used Au/TiO_2_ (Figure S13) [40, 43, 50] exhibits very low activity with the selectivity of 75.9% (entry 16). Blank experiments without catalyst or use of Pt-free MoC show no conversion further confirming the indispensable role of Pt species for the desired transformation.

To examine whether the Pt-catalyzed reaction indeed occurred on the surface of Pt_1_/MoC, the solid was removed from the reaction system after 2 h. Postprocessing of the filtrate under identical conditions for another 2 h did not increase the conversion. Furthermore, the inductively coupled plasma atomic emission spectroscopy (ICP-AES) data of the filtrate shows that the content of Pt or Mo in the solution is below the detection limit of 0.1 ppm, indicating the leaching is negligible during the reaction and the heterogeneous catalysis nature of Pt_1_/MoC. The apparent activation energy (*E*_a_) was estimated to be 32.42 kJ mol^−1^ (Figure S14), which is lower than those of the existing catalysts [42–44, 47–49]. Pt_1_/MoC can be reused at least for six runs without significant decay (Figure S15), indicative of the excellent stability for quinoline transformation. Compared to the fresh Pt_1_/MoC, no visible changes in the morphology, single crystal nature, and surface chemical state of Pt species of the used Pt_1_/MoC were observed and no aggregation of the Pt single atoms occurred (Figures S8, S9, S10, and S16–18 and Table S2). These results demonstrate the effectiveness of Pt_1_/MoC catalyst for selective quinoline hydrogenation.

Moreover, Pt_1_/MoC was extended to the investigation of various structurally different substituted quinoline compounds, as listed in Table 2. Reactions involving a methyl group at the 2- and 4-position proceeded smoothly to produce the corresponding 1,2,3,4-tetrahydroquinolines (Table 2, entries 1 and 2). The 2-position substrate was easily hydrogenated with higher average TOF of 2102 h^−1^ (initial TOF of 2522 h^−1^). Low performance for 2-methyl-4-hydroxyquinoline was obtained (entry 3) probably due to both steric and electronic effects [42, 50, 51]. For more challenging reaction involving reducible halogen groups (e.g., -Cl and -F), Pt_1_/MoC shows significant activities without any dehalogenation (entries 4 and 5). Interestingly, Pt_1_/MoC achieves the hydrogenation of isoquinoline with high selectivity (>99%) and moderate conversion (81.3%) (entry 6). Furthermore, Pt_1_/MoC could conduct the tentative experiment of heteroaromatic indole reduction, an important process in pharmaceutical synthesis [52], in spite of the low efficiency (entry 7).

**Figure 3:**
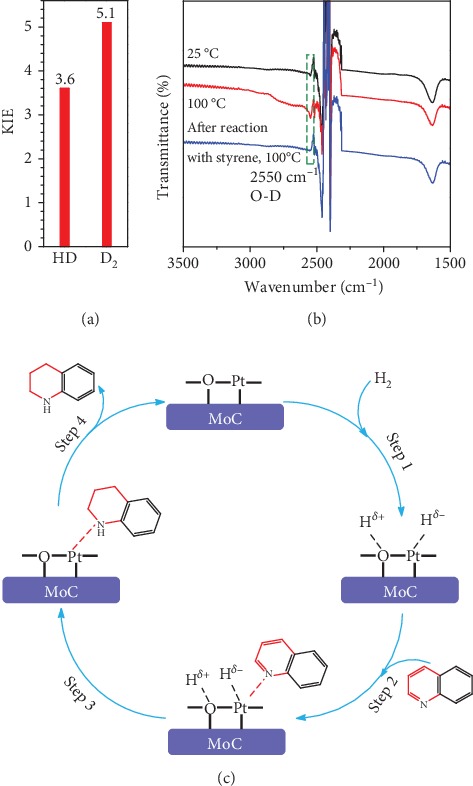
(a) KIEs of selective quinoline hydrogenation in the presence of HD or D_2_ using Pt_1_/MoC. (b) Quasi *in situ* FTIR spectra of Pt_1_/MoC for the chemical activation of D_2_ at 25 and 100°C. The O–D vibration disappears after the introduction of styrene. (c) Possible reaction pathway for selective hydrogenation of quinoline to 1,2,3,4-tetrahydroquinoline over Pt_1_/MoC catalyst.

The correlation between Pt_1_–MoC interactions and performance of Pt_1_/MoC catalyst for quinoline hydrogenation was further studied to gain insights into the reaction mechanism. Primary kinetic isotope effects (KIEs) with H_2_ being labeled using HD or D_2_ are summarized in Table S4 and Figure 3(a). HD and D_2_ show significant KIEs in the reduction process, and the latter exhibits larger value of 5.1. These results indicate that the cleavage of H-H bond and the formation of C-C and C-N bonds in pyridine ring are kinetically relevant steps in quinoline hydrogenation [47]. In order to probe the potential key intermediates under the presence of D_2_ over surface positively charged Pt_1_/MoC, quasi *in situ* Fourier transform infrared (FTIR) spectroscopy was conducted. The IR vibration of O–D bond at 2550 cm^−1^ was clearly observed (Figure 3(b)), and the heterolytic dissociation of H_2_ was promoted by temperature, forming O–D(*δ*^+^) and Pt–D(*δ*^–^) species. Besides the intrinsic effect of the atomically dispersed Pt species, MoC as a conductor can also facilitate the H_2_ activation and dissociation [37], and the suitable Pt–MoC interactions are beneficial for these key steps. Note that the rate of H_2_ activation and dissociation was much faster than that of the overall process of hydrogenation [43, 47]. Thus, the rate-determining step (RDS) can be derived from the subsequent hydrogenation with one hydrogen transfer from Pt–H(*δ*^–^) and another from O–H(*δ*^+^) to the pyridine ring.

Based on the catalytic performance, KIEs, and FTIR results, a possible reaction pathway for selective quinoline hydrogenation over Pt_1_/MoC was proposed (Figure 3(c)). The hydrogenation process may consist of the following four steps: (i) initial adsorption and chemical activation of H_2_ molecules over the positively charged Pt with heterolytic dissociation forming Pt–H(*δ*^–^) and O–H(*δ*^+^) species; (ii) chemisorption of quinoline molecules on the catalyst surface primarily through the Pt-N interaction, thus facilitating the activation of pyridine ring; (iii) generation of the adsorbed 1,2,3,4-tetrahydroquinoline *via* the transfer of H(*δ*^–^) and H(*δ*^+^) intermediates to the activated pyridine ring; and (iv) desorption of the goal product 1,2,3,4-tetrahydroquinoline from the catalyst surface. The adsorption and/or chemical activation of H_2_ and quinoline molecules are widely considered to be the kinetically relevant step and pyridine ring hydrogenation as the RDS [47]. Although considerable amount of energy is required for quinoline hydrogenation, the appropriate interaction of Pt-N (Pt_1_/MoC-quinoline) and the strong interactions between positively charged Pt atoms and MoC substrate could efficiently surpass the total reaction barriers and achieve the formation of desired 1,2,3,4-tetrahydroquinoline.

Pt_1_/MoC was further examined for selective hydrogenation of *α*,*β*-unsaturated aldehyde involving C=C and C=O groups [39, 53–57]. The SAC exhibits a high efficiency with the average TOF of 1216 h^−1^ for crotonaldehyde hydrogenation (Table S5). This is the best performance reported for crotonaldehyde hydrogenation via heterogeneous catalysis under identical conditions and is comparable with most values gained using organic complexes [53–55]. Similar to quinoline hydrogenation, crotonaldehyde conversion using Pt NPs leads to less efficiencies (Table S5). These results again demonstrate the remarkable benefit of using single-atom Pt catalyst for selective reduction of *α*,*β*-unsaturated aldehyde and further suggest the broad application prospects of Pt SAC in fine chemical synthesis.

## 3. Conclusion

A facile and controllable one-step arc-discharge strategy at ultrahigh temperature was successfully developed to synthesize single-atom Pt catalyst. High-temperature-stabilized Pt_1_/MoC with a unique electronic/geometric structure exhibits outstanding performance and excellent thermal stability for selective quinoline hydrogenation. The activity of 3710 h^−1^ is better than those of previously reported catalysts by an order of magnitude under similar conditions. Pt_1_/MoC shows broad scope activity toward hydrogenation including quinoline compounds and crotonaldehyde containing C=C, C=N, and C=O groups. Primary kinetic isotope effects and *in situ* FTIR analysis show that the cleavage of H-H bond and the formation of C-C and C-N bonds in pyridine ring over the positively charged Pt_1_/MoC are kinetically relevant steps and the latter is RDS. The possible four-step mechanism involving the heterolytic dissociation of H_2_ and the transfer of H(*δ*^−^) and H(*δ*^+^) species to the activated pyridine ring *via* Pt-N bond over Pt_1_/MoC is proposed. We anticipate that the one-step high-temperature strategy will allow the development of broad SACs for the important yet challenging chemical transformations.

## 4. Materials and Methods

### 4.1. Preparation of MoC

The MoC was synthesized by a direct current arc-discharge method (Figures 1 and S1) [45, 46]. The cathode was a pure graphite rod with a diameter of 8 mm. The anode was a graphite tube with an external diameter of 8 mm and an inner diameter of 6 mm. The graphite tube was filled with Mo powders. The two electrodes were installed horizontally, and the cathode was fixed on a water-cooled copper pedestal. The arc chamber was first evacuated to 3 Pa and then filled by pure H_2_ to the pressure of 0.08 MPa. The arc was generated at a current of 80 A, and the distance between the two electrodes was kept at about 2 mm by physically adjusting the cathode. The typical synthesis time was about 30 min, and about 1 cm anode was consumed. After reaction, the powders generated on the top of the arc chamber were collected.

### 4.2. Preparation of Pt/MoC Catalysts

In analogy to the case with MoC, the Pt/MoC catalysts were one-step *in situ* prepared by the arc-discharge method. The graphite tube, which was filled with a mixture of Pt and Mo powders, was used as anode. The mass fraction of Pt in the mixture depends on the mass loading of Pt in the final Pt/MoC product. By controlling the Pt loading, the Pt_1_/MoC can be easily achieved.

### 4.3. Catalyst Characterization

XRD characterization of the samples was carried out on a German Bruker D8 Advance X-ray diffractometer using the Ni-filtered Cu K*α* radiation at 40 kV and 40 mA. XPS data were recorded with the Axis Ultra Photoelectron Spectrometer (Kratos Analytical Ltd.) by means of a monochromatized Al K*α* anode (225 W, 15 mA, 15 kV). The C 1s peak at 284.8 eV was used as the reference to calibrate the binding energies (BE). A Tecnai F20 electron microscope operating at 200 kV equipped with an EDS unit (Si(Li) detector) was used for the TEM investigations. The samples for electron microscopy were prepared by grinding and subsequent dispersing the powder in ethanol and applying a drop of very dilute suspension on carbon-coated grids. High resolution TEM, EDS mapping, and HADDF-STEM were performed on an FEI Titan G2 (60-300) probe-corrected TEM system with a field emission gun operated at 300 kV.

### 4.4. Catalytic Activity Test

The selective hydrogenations were carried out in a high-pressure stainless autoclave reactor (Parr Instrument Co., 4790, 50 mL). Typically, 2 mL solvent (toluene or ethanol), a certain amount of substrate (quinoline or quinoline compounds or crotonaldehyde), and a known amount of catalyst were placed in the autoclave. The autoclave was sealed and flushed several times with 0.5 MPa H_2_ to remove the air in the reactor; then, 2 MPa H_2_ was charged. The stirrer (800 rpm) was started until the desired temperature was reached. After a certain time, the autoclave was placed in cool water and the gas was carefully released. The gaseous mixture was analyzed using a gas chromatograph (GC) Agilent 7820A equipped with a TDX-01 column connected to a thermal conductivity detector. A known amount of internal standard 1,4-dioxane was added into the aqueous product in autoclave. The reaction mixture was transferred into a centrifuge tube, and the solid catalyst was separated by centrifugation. The product solution was quantitatively analyzed using a GC Agilent 7820A equipped with a HP-5 capillary column connected to a flame ionization detector. Identification of the products was performed by using a GC-MS spectrometer. Noted that the total carbon balance was >95%. For the recycling experiment, the centrifuged catalysts from parallel tests were collected and washed with distilled water several times, followed by air drying at 120°C for 12 h. The KIE experiments were performed by following the same procedure as selective hydrogenations. The H_2_ or HD or D_2_ was, respectively, used as hydrogen source. The products were qualitatively and quantitatively analyzed using a GC and GC-MS spectrometer. The conversions of quinoline were kept below 20% for calculation of initial TOF. The unit of TOF is h^−1^, that is, mol target product per mol noble metal site per hour.

## Figures and Tables

**Table 1:** Study of various solid catalysts for the selective hydrogenation of quinoline^a^.

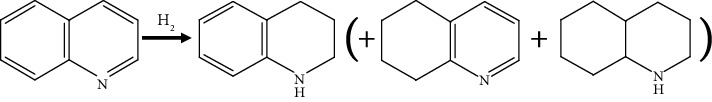					
Entry	Catalyst	Conv. (%)	Sel. (%)	TOF (h^−1^)^b^	TOF (h^−1^)^c^
1^d^	2% Pt/MoC	>99	>99	393	2038
2	1% Pt/MoC	95.8	>99	766	2465
3^e^	0.5% Pt/MoC	76.6	>99	1225	2792
4^f^	0.2% Pt/MoC	96.3	>99	1921	3143
5^g^	Pt_1_/MoC	84.5	>99	3380	3710
6^g,h^	Pt_1_/MoC	>99	>99	1975	3687
7^h,i^	1% Pt/MoC	54.8	>99	219	815
8^j^	1% Pt/MoC	61.7	>99	494	1630
9	1% Pt/NbC	75.9	95.6	582	—
10	1% Pt/WC	42.6	92.7	318	—
11	1% Pt/TiC	56.8	94.9	433	—
12	1% Pd/MoC	81.3	72.5	470	—
13	1% Ru/MoC	85.2	90.6	623	—
14	1% Au/MoC	66.2	98.1	525	—
15^k^	1% Pt/MoC	82.5	>99	659	—
16	1% Au/TiO_2_	34.7	75.9	216	—

^a^Reaction conditions: 0.025 mol% metal, 2 mL toluene, 4.15 mmol quinoline, 2 MPa H_2_, 100°C, 5 h. ^b^Average TOF based on total metal sites. ^c^TOF based on surface Pt sites and 20% quinoline conversion. ^d^0.05 mol% Pt. ^e^0.0125 mol% Pt. ^f^0.01 mol% Pt. ^g^0.005 mol% Pt. ^h^10 h. ^i^60°C. ^j^1 MPa H_2_. ^k^1% Pt/MoC prepared by conventional impregnation route.

**Table 2:** Pt_1_/MoC-catalyzed selective hydrogenation of quinoline compounds^a^.

Entry	Substrate	Target product	Conv. (%)	Sel. (%)	TOF (h^−1^)^b^	TOF (h^−1^)^c^
1^d^	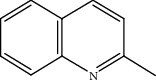	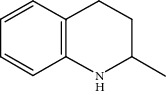	53.1	>99	2102	2522
2	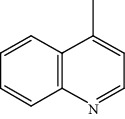	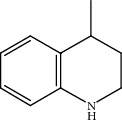	68.3	>99	1352	1690
3	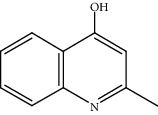	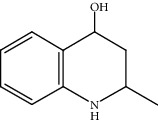	45.8	>99	906	1178
4	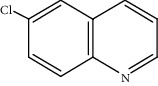	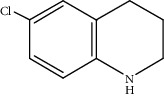	86.5	>99	1712	2054
5	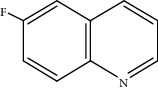	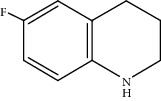	63.9	96.7	1235	1359
6	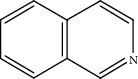	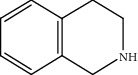	81.3	>99	1609	1908
7	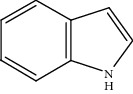	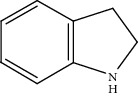	33.8	45.6	308	335

^a^Reaction conditions: 0.005 mol% Pt, 5 mL toluene, 3 mmol substrate, 2 MPa H_2_, 150°C, 10 h. ^b^Average TOF. ^c^TOF based on 20% substrate conversion. ^d^5 h.

## Appendix

Table of content.

## Supplementary Materials

Supplementary Materials Figure S1: photograph of high-temperature arc-discharge facility. The facility consists of four main parts of vacuum system, control system, water-cooled system, and arc-discharge chamber. Figure S2: summary of the results of single-atom catalyst synthesis temperatures and time by one-step high-temperature arc-discharge strategy and other techniques (wet chemical, atomic layer deposition, furnace annealing, and shockwave) in the literature. Figure S3: N2 adsorption-desorption isotherms of MoC sample. Table S1: properties of MoC and Pt/MoC catalysts. Figure S4: XRD patterns of MoC and Pt/MoC samples. The absence of the dominant Pt(111) crystal phase at 2 theta of 39.4o even with the loading content of Pt as high as 2 wt% indicates the high dispersion of the Pt species. Figure S5: HRTEM images of 1% Pt/MoC catalyst. Figure S6: (a) TEM image and (b) HAADF-STEM-EDS elemental mappings of Mo, C, and Pt of Pt1/MoC catalyst. Figure S7: aberration-corrected STEM-HAADF image of Pt1/MoC catalyst. Atomically dispersed Pt atoms are highlighted by the red circles. Figure S8: EXAFS Fourier transform moduli at Pt *L*_3_-absorption edge of (a) 0.2% Pt/MoC, (b) 1% Pt/MoC, (c) 2% Pt/MoC, and (d) used Pt1/MoC samples. Table S2: EXAFS parameters of Pt/MoC samples. Figure S9: data and imaginary portion of the FT-EXAFS spectra of (a) Pt1/MoC, (b) used Pt1/MoC, (c) 0.2% Pt/MoC, (d) 1% Pt/MoC, and (e) 2% Pt/MoC samples. Figure S10: Pt 4f XPS data of (a) 0.2% Pt/MoC, (b) 0.5% Pt/MoC, (c) 1% Pt/MoC, and (d) used Pt1/MoC. Table S3: comparison of selective hydrogenation of quinoline. Figure S11: HRTEM images of (a) 1% Pt/NbC, (b) 1% Pt/WC, (c) 1% Pt/TiC, (d) 1% Pd/MoC, (e) 1% Ru/MoC, and (f) 1% Au/MoC catalysts. Figure S12: HRTEM images of 1% Pt/MoC catalyst prepared using the conventional impregnation method. Figure S13: HRTEM image of 1% Au/TiO_2_ catalyst. Figure S14: Arrhenius plot for Pt1/MoC catalyzed selective hydrogenation of quinoline. Reaction conditions: 0.005 mol% Pt, 2 mL toluene, 4.15 mmol quinoline (0.5 mL), 2 MPa H_2_, 60–100°C, 1 h. The conversions were kept below 20% for calculation of initial TOF. Figure S15: recycling of Pt1/MoC catalyst for selective hydrogenation of quinoline. Reaction conditions for each run: 0.01 mol% Pt, 2 mL toluene, 4.15 mmol quinoline (0.5 mL), 2 MPa H_2_, 100°C, 10 h. Figure S16: (a) HAADF-STEM and (b) aberration-corrected STEM-HAADF images of the used Pt1/MoC catalyst. Inset in (b) is the FFT data showing the single-crystal structure of the used Pt1/MoC. Atomically dispersed Pt atoms in image (b) are highlighted by the red circles. Figure S17: (a) TEM image and (b) HAADF-STEM-EDS elemental mappings of Mo, C, and Pt of the used Pt1/MoC catalyst. Figure S18: aberration-corrected STEM-HAADF image of the used Pt1/MoC catalyst. Atomically dispersed Pt atoms are highlighted by the red circles. Table S4: kinetic isotope effects of selective hydrogenation of quinoline using Pt1/MoC. Table S5: study of various solid catalysts for the selective hydrogenation of crotonaldehyde.
