# Supplementary material for: One-Step High-Temperature-Synthesized Single-Atom Platinum Catalyst for Efficient Selective Hydrogenation
Source: Research (Wash D C). 2020 Apr 29;2020:9140841. doi: 10.34133/2020/9140841 (PMC7206892; doi:10.34133/2020/9140841)
Supplement: Supplementary Materials — Figure S1: photograph of high-temperature arc-discharge facility. The facility consists of four main parts of vacuum system, control system, water-cooled system, and arc-discharge chamber. Figure S2: summary of the results of single-atom catalyst synthesis temperatures and time by one-step high-temperature arc-discharge strategy and other techniques (wet chemical, atomic layer deposition, furnace annealing, and shockwave) in the literature. Figure S3: N2 adsorption-desorption isotherms of MoC sample. Table S1: properties of MoC and Pt/MoC catalysts. Figure S4: XRD patterns of MoC and Pt/MoC samples. The absence of the dominant Pt(111) crystal phase at 2 theta of 39.4o even with the loading content of Pt as high as 2 wt% indicates the high dispersion of the Pt species. Figure S5: HRTEM images of 1% Pt/MoC catalyst. Figure S6: (a) TEM image and (b) HAADF-STEM-EDS elemental mappings of Mo, C, and Pt of Pt1/MoC catalyst. Figure S7: aberration-corrected STEM-HAADF image of Pt1/MoC catalyst. Atomically dispersed Pt atoms are highlighted by the red circles. Figure S8: EXAFS Fourier transform moduli at Pt L3-absorption edge of (a) 0.2% Pt/MoC, (b) 1% Pt/MoC, (c) 2% Pt/MoC, and (d) used Pt1/MoC samples. Table S2: EXAFS parameters of Pt/MoC samples. Figure S9: data and imaginary portion of the FT-EXAFS spectra of (a) Pt1/MoC, (b) used Pt1/MoC, (c) 0.2% Pt/MoC, (d) 1% Pt/MoC, and (e) 2% Pt/MoC samples. Figure S10: Pt 4f XPS data of (a) 0.2% Pt/MoC, (b) 0.5% Pt/MoC, (c) 1% Pt/MoC, and (d) used Pt1/MoC. Table S3: comparison of selective hydrogenation of quinoline. Figure S11: HRTEM images of (a) 1% Pt/NbC, (b) 1% Pt/WC, (c) 1% Pt/TiC, (d) 1% Pd/MoC, (e) 1% Ru/MoC, and (f) 1% Au/MoC catalysts. Figure S12: HRTEM images of 1% Pt/MoC catalyst prepared using the conventional impregnation method. Figure S13: HRTEM image of 1% Au/TiO2 catalyst. Figure S14: Arrhenius plot for Pt1/MoC catalyzed selective hydrogenation of quinoline. Reaction conditions: 0.005 mol% Pt, 2 mL toluene, 4.15 m [file 9140841.f1.doc]

**Supporting Information for**

**One-Step High-Temperature Synthesized Single-Atom Platinum Catalyst for Efficient Selective Hydrogenation**

Qingyuan Bi,^1^ Xiaotao Yuan,^2^ Yue Lu,^3^ Dong Wang,^1^ Jian Huang,^1^ Rui Si,^4^* Manling Sui,^3^* and Fuqiang Huang^1,2^*

^1^ State Key Laboratory of High Performance Ceramics and Superfine Microstructure, Shanghai Institute of Ceramics, Chinese Academy of Sciences, Shanghai 200050, P. R. China

^b^State Key Laboratory of Rare Earth Materials Chemistry and Applications, College of Chemistry and Molecular Engineering, Peking University, Beijing 100871, P. R. China

^c^Institute of Microstructure and Properties of Advanced Materials, Beijing University of Technology, Beijing 100124, P. R. China

^d^Shanghai Synchrotron Radiation Facility, Shanghai Institute of Applied Physics, Chinese Academy of Sciences, Shanghai 201800, P. R. China

Corresponding Authors

*sirui@sinap.ac.cn

*mlsui@bjut.edu.cn

*huangfq@mail.sic.ac.cn

**1. Chemicals and materials**

Quinoline (C_9_NH_7_, 99 wt%), 1,2,3,4-tetrahydroquinoline (C_9_NH_11_, 97 wt%), 5,6,7,8-tetrahydroquinoline (C_9_NH_11_, 98 wt%), decahydroquinoline (C_9_NH_17_, 98 wt%), toluene (C_7_H_8_, 99.5 wt%), 2-methylquinoline (C_10_NH_9_, 98 wt%), 2-methyl-1,2,3,4-tetrahydroquinoline (C_10_NH_13_, 98 wt%), 4-methylquinoline (C_10_NH_9_, 98 wt%), 4-methyl-1,2,3,4- tetrahydroquinoline (C_10_NH_13_, 97 wt%), 2-methyl-4-hydroxyquinoline (C_10_NH_9_O, 98 wt%), 2-methyl-4-hydroxy- 1,2,3,4-tetrahydroquinoline (C_10_NH_13_O, 98 wt%), 6-chloroquinoline (C_9_NH_6_Cl, 98 wt%), 6-chloro-1,2,3,4-tetra- hydroquinoline (C_9_NH_10_Cl, 98 wt%), 6-fluoroquinoline (C_9_NH_6_F, 98.5 wt%), 6-fluoro-1,2,3,4-tetrahydroquinoline (C_9_NH_10_F, 98 wt%), isoquinoline (C_9_NH_7_, 97 wt%), 1,2,3,4-tetrahydroisoquinoline (C_9_NH_11_, 98 wt%), indole (C_8_NH_7_, 99 wt%), crotonaldehyde (C_4_H_6_O, 98 wt%), crotyl alcohol (C_4_H_8_O, 99 wt%), butyl aldehyde (C_4_H_8_O, 99 wt%), butanol (C_4_H_10_O, 98 wt%), styrene (C_8_H_8_, 99 wt%), chloroplatonic acid hexahydrate (H_2_PtCl_6_·6H_2_O), chloroauric acid tetrahydrate (HAuCl_4_·4H_2_O), sodium hydroxide (NaOH, 99.5 wt%), Mo powder (99.95%), Pt powder (99.9%), Pd powder (99.9%), Ru powder (99.9%), Au powder (99.9%), Nb powder (99.9%), W powder (99.9%), and Ti powder (99.9%) were supplied by Alfa Aesar and used without further purification.

D_2_ (> 97%) and HD (90%) were obtained from Shanghai Research Institute of Chemical Industry CO., LTD.

TiO_2_ (P25, specific surface area: 45 m^2^ g^–1^, 70% anatase and 30% rutile) was purchased from Evonik.

**2. Catalyst preparation**

**2.1 Preparation of 1% Pt/NbC, 1% Pt/WC, and 1% Pt/TiC catalysts:** All these catalysts were prepared using the similar arc-discharge route. The graphite tube, which was filled with a mixture of Pt and Nb or W or Ti powders, was used as anode.

**2.2 Preparation of 1% Pd/MoC, 1% Ru/MoC, and 1% Au/MoC catalysts:** All these catalysts were prepared using the similar arc-discharge route. The graphite tube, which was filled with a mixture of Pd or Ru or Au and Mo powders, was used as anode.

**2.3 Preparation of 1% Pt/MoC with impregnation method:** The catalyst was prepared using the routine incipient wetness technique [S1]. Appropriate amount of MoC (1.0 g as-prepared MoC sample) was added to the aqueous solution of H_2_PtCl_6_·6H_2_O. After a perfect mixing of the corresponding slurries and static standing for 12 h, the sample was dried at 100 ^o^C for 12 h, followed by reduction in 5 vol% H_2_/Ar at 350 ^o^C (ramping rate of 5 ^o^C min^–1^) for 2 h.

**2.4 Preparation of 1% Au/TiO_2_ catalyst:** The 1% Au/TiO_2_ catalyst was prepared by using conventional deposition-precipitation (DP) method [S2]. Briefly, an appropriate amount of TiO_2_ (P25) was added to the aqueous solution of HAuCl_4_ (1 mM) at a fixed pH (9.0) adjusted with NaOH (0.2 M). After 6 h stirring at room temperature the catalyst was washed five times with deionized water and separated by filtration. The sample was dried at 100 ^o^C in air for 5 h, followed by a careful reduction with a stream of 5 vol% H_2_/Ar at 350 ^o^C (ramping rate of 5 ^o^C min^–1^) for 2 h.

**3. Catalyst characterization**

**3.1 Elemental analysis:** The metal loading of the catalysts was measured by inductively coupled plasma atomic emission spectroscopy (ICP-AES) using a Thermo Electron IRIS Intrepid II XSP spectrometer.

**3.2 BET analysis:** The BET specific surface areas of the prepared catalysts were determined by adsorption– desorption of nitrogen at liquid nitrogen temperature, using a Micromeritics ASAP 2460 equipment. Sample degassing was carried out at 300 ^o^C prior to acquiring the adsorption isotherm.

**3.3 Diffuse Reflectance Infrared Fourier Transform (DRIFT) measurements:** The CO adsorption experiments were carried out on a Bruker Vector 22 FTIR spectrometer equipped with a MCT detector and Harrick diffuse reflectance accessory. Spectra were obtained on the apparatus loaded with 50 mg of catalyst. Prior to the saturated adsorption of 0.5 vol % CO/He at room temperature, sample was subjected to the pretreatment with Helium flow at 200 ^o^C for removing any other gases and moisture. Each spectrum was obtained after the 10 min evacuation treatment with Helium flow at room temperature and by subtracting the background (base spectrum) of the unloaded sample.

**3.4 Quasi *in situ* Fourier Transform Infrared (FTIR) measurements:** The quasi *in situ* FTIR measurements were conducted on a Shimadzu IRPrestige-21 FTIR spectrometer equipped with a DLATGS detector. To characterize the formation of the O–D bond when Pt_1_/MoC was exposed to D_2_, the wafer catalyst was first placed into a homemade closed chamber (200 mL) with a quartz light-transmission system. After being degassed with Ar flow for 30 min at room temperature, the background was recorded, and the chamber containing Pt_1_/MoC catalyst was then filled with sufficient D_2_. After reaction for 1 h, the spectrum was detected. Then, the chamber was heated at 100 ^o^C for 30 min, and the spectrum was detected. Later, a certain amount of styrene was quickly injected into the chamber at 100 ^o^C, and the spectrum was detected after 10 min.

**3.5 X-ray absorption fine structure (XAFS):** The XAFS spectra at Pt *L*_3_ (*E*_0_ = 11564 eV) edge were performed at BL14W1 beamline of Shanghai Synchrotron Radiation Facility (SSRF) operated at 3.5 GeV under “top-up” mode with a constant current of 240 mA. The XAFS data were recorded under fluorescence mode with Si(111) monochromator and 7-element Ge solid state detector or Lytle ion chamber. The energy was calibrated accordingly to the absorption edge of pure Pt foil. Athena and Artemis codes were used to extract the data and fit the profiles. For the X-ray absorption near edge spectroscopy (XANES) part, the experimental absorption coefficients as function of energies *μ*(*E*) were processed by background subtraction and normalization procedures, and reported as “normalized absorption”. For the extended X-ray absorption fine structure (EXAFS) part, the Fourier transformed (FT) data in R space were analyzed by applying first shell approximate and Pt metal models for Pt-O and Pt-Pt contributions, respectively. The parameters describing the electronic properties (e.g., correction to the photoelectron energy origin, *E*_0_) and local structure environment including *CN*, distance (*R*) and Debye-Waller factor around the absorbing atoms were allowed to vary during the fit process.

**3.6 CO chemisorption measurements:** Pt surface sites were measured by CO chemisorptions using AutoChem HP 2950 apparatus with quantitative loop from Micromeritics. The sample was first pretreated with 5 vol % H_2_/Ar at 400 ^o^C for 2 h, and then pure CO was pulsed over the sample at 25 ^o^C for several times to saturated adsorption. The total amount of CO adsorbed was measured by assuming a chemisorption stoichiometry of CO/Pt=1 and a Pt surface atomic density of 1.25×10^19^ m^-2^. The dispersion (D) was calculated according to the formula of D = (surface number of Pt atoms)/(total number of Pt atoms).


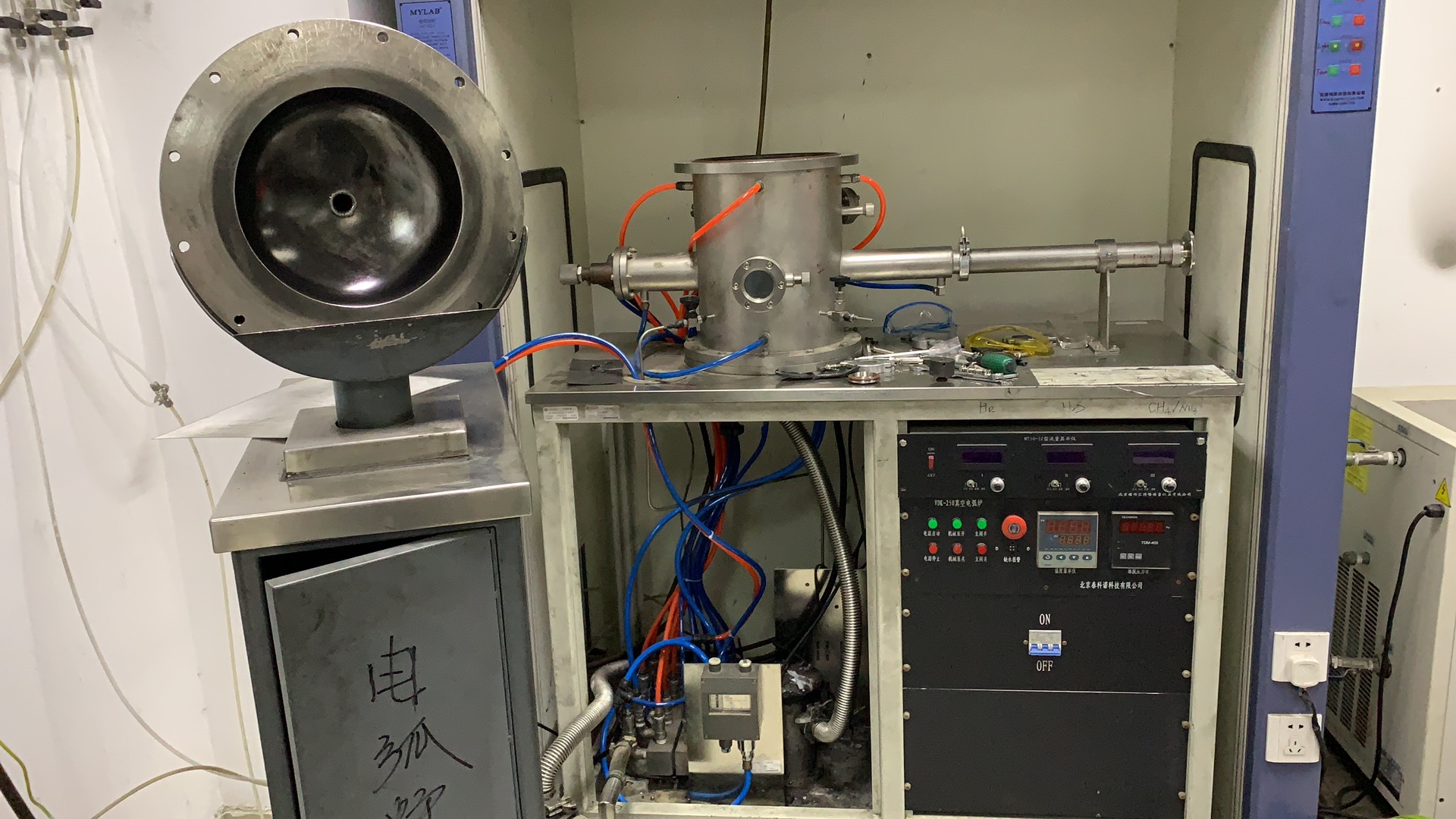


**Figure S1.** Photograph of high-temperature arc discharge facility. The facility consists of four main parts of vacuum system, control system, water-cooled system, and arc discharge chamber.

**Figure S2.** Summary of the results of single-atom catalyst synthesis temperatures and time by one-step high-temperature arc discharge strategy and other techniques (wet chemical [S1, S3−S6], atomic layer deposition [S7], furnace annealing [S8], and shockwave [S9]) in the literature.

**Figure S3.** N_2_ adsorption-desorption isotherms of MoC sample.

**Table S1.** Properties of MoC and Pt/MoC catalysts.

| Sample | Mo/C molar ratio^a^ | Pt loading (wt%)^b^ | BET surface area (m^2^ g^–1^) | Pt particle size  (nm)^c^ |
| --- | --- | --- | --- | --- |
| MoC | 1/0.85 | – | 65 | – |
| 2% Pt/MoC | 1/0.86 | 1.98 | 56 | 3.1 ± 0.2 |
| 1% Pt/MoC | 1/0.83 | 1.05 | 61 | 2.2 ± 0.2 |
| 0.5% Pt/MoC | 1/0.83 | 0.48 | 59 | 1.3 ± 0.1 |
| 0.2% Pt/MoC | 1/0.82 | 0.22 | 68 | 1.1 ± 0.1 and  partial atomic dispersion |
| Pt_1_/MoC | 1/0.85 | 0.11 | 62 | Atomic dispersion |

^a^ According to the elemental analysis. ^b^ According to the ICP-AES analysis. ^c^ Average diameter.

**Figure S4.** XRD patterns of MoC and Pt/MoC samples. The absence of the dominant Pt(111) crystal phase at 2 theta of 39.4^o^ even with the loading content of Pt as high as 2 wt% indicates the high dispersion of the Pt species.

**Figure S5.** HRTEM images of 1% Pt/MoC catalyst.

**Figure S6.** (a) TEM image and (b) HAADF-STEM-EDS elemental mappings of Mo, C, and Pt of Pt_1_/MoC catalyst.

**Figure S7.** Aberration-corrected STEM-HAADF image of Pt_1_/MoC catalyst. Atomically dispersed Pt atoms are highlighted by the red circles.

**Figure S8.** EXAFS Fourier transform moduli at Pt *L_3_*-absorption edge of (a) 0.2% Pt/MoC, (b) 1% Pt/MoC, (c) 2% Pt/MoC, and (d) used Pt_1_/MoC samples.

**Table S2.** EXAFS parameters of Pt/MoC samples.

| Sample | Shell | *N* | *R* (Å) | *σ*^2^ (×10^–3^ Å^2^) | Δ*E*_0_ (eV) |
| --- | --- | --- | --- | --- | --- |
| Pt foil | Pt-Pt | 12.0 | 2.764 ± 0.002 | 4.9 ± 0.2 | 9.1 ± 0.6 |
| Pt_1_/MoC | Pt-O | 1.8 ± 0.6 | 2.00 ± 0.02 | 5.0 ± 0.2 | 10.3 ± 0.9 |
|  | Pt-Pt |  |  |  |  |
| used Pt_1_/MoC | Pt-O | 1.7 ± 0.6 | 2.03 ± 0.02 | 5.0 ± 0.2 | 10.3 ± 0.9 |
|  | Pt-Pt |  |  |  |  |
| 0.2% Pt/MoC | Pt-O | 0.8 ± 0.7 | 2.00 ± 0.06 | 5.0 ± 0.2 | 10.3 ± 0.9 |
|  | Pt-Pt | 1.8 ± 0.5 | 2.74 ± 0.03 | 6.0 ± 0.3 | 10.3 ± 0.9 |
| 1% Pt/MoC | Pt-O | 1.8 ± 0.2 | 2.04 ± 0.01 | 5.0 ± 0.2 | 10.3 ± 0.9 |
|  | Pt-Pt | 1.8 ± 0.5 | 2.75 ± 0.01 | 6.0 ± 0.3 | 10.3 ± 0.9 |
| 2% Pt/MoC | Pt-O | 1.4 ± 0.1 | 2.04 ± 0.01 | 5.0 ± 0.2 | 10.3 ± 0.9 |
|  | Pt-Pt | 1.8 ± 0.5 | 2.76 ± 0.01 | 6.0 ± 0.3 | 10.3 ± 0.9 |

*N*, coordination number; *R*, distance between absorber and backscatter atoms; *σ*^2^, the Debye-Waller factor value; Δ*E*_0_, inner potential correction to account for the difference in the inner potential between the sample and the reference compound.

**Figure S9.** Data and imaginary portion of the FT-EXAFS spectra of (a) Pt_1_/MoC, (b) used Pt_1_/MoC, (c) 0.2% Pt/MoC, (d) 1% Pt/MoC, and (e) 2% Pt/MoC samples.

**Figure S10.** Pt 4f XPS data of (a) 0.2% Pt/MoC, (b) 0.5% Pt/MoC, (c) 1% Pt/MoC, and (d) used Pt_1_/MoC.

**Table S3.** Comparison of selective hydrogenation of quinoline.

| Catalyst | Conditions | Conv./Sel. (%) | TOF (h^−1^)^a^ | Ref. |
| --- | --- | --- | --- | --- |
| Pt_1_/MoC | 4.15 mmol quinoline, 2 mL toluene, S/C=20000, 100 ^o^C, 2 MPa H_2_, 5 h | 99/99 | 3380 | This work |
| Ru/HAP | 23.4 mg, 90 ^o^C, S/C=300, 3 MPa H_2_, 2 mL EtOH, 1 h | 92.2/96.2 | 266 | [S10] |
| 1.5 nm Pd^0^HAP(30) | 0.5 mmol quinoline, 6 mL toluene, S/C= 250, 1 bar H_2_, 50 ^o^C, 4.5 h | 98/98 | 53 | [S11] |
| Pd-BT-CF | 6 μmol Pd, 1 mmol quinoline, 3 mL H_2_O, 2 MPa H_2_, 80 ^o^C, 1 h | 99.9/100 | 167 | [S12] |
| Au/HSA-TiO_2_ | 3 mL toluene, 0.5 mmol quinoline, S/C=100, 2 MPa H_2_, 60 ^o^C, 3.5 h | 100/100 | 28 | [S13] |
| Pd@ompg-C_3_N_4_ | 0.5 mmol quinoline, 5 mL acetonitrile, S/C= 21, 1 bar H_2_, 30 ^o^C, 4 h | 99/99 | 5 | [S14] |
| BWT-Pd | 50 μmol Pd, 15 mg BWT, 5 mmol quinoline, 3 mL n-BuOH, 2 MPa H_2_, 90 ^o^C, 30 min | 98/100 | 196 | [S15] |
| AuNPore | 0.5 mmol quinoline, 1.25 mmol PhMe_2_SiH, 1.5 mmol H_2_O, 0.5 mL toluene, S/C= 50, 80 ^o^C, 24 h | 91 of yield | 2 | [S16] |
| Au/TiO_2_-R | 0.5 mmol quinoline, S/C=100, FA/ Et_3_N/DMF, 130 ^o^C, 10 min | 99/96 | 570 | [S17] |
| Ru/NHPC | 25 mg cat., 5 mmol quinoline, 5 mL EtOH, 1 MPa H_2_, 100 ^o^C, 1 h | 97/98 | 654 | [S18] |
| SiO_2_@RF/Pt-1.2 nm | 0.2 mmol quinoline, 2 mL toluene, S/C=20, 25 ^o^C, 1 bar H_2_, 80 min | 99/99 | 15 | [S19] |
| Single Ru/N-C | 0.1 mmol quinoline, 3 mL THF, S/C=100, 100 ^o^C, 3.5 MPa H_2_, 4 h | 99/99 | 25 | [S20] |

^a^ Average TOF based on the conversion of quinoline.

**Figure S11.** HRTEM images of (a) 1% Pt/NbC, (b) 1% Pt/WC, (c) 1% Pt/TiC, (d) 1% Pd/MoC, (e) 1% Ru/MoC, and (f) 1% Au/MoC catalysts.

**Figure S12.** HRTEM images of 1% Pt/MoC catalyst prepared using conventional impregnation method.


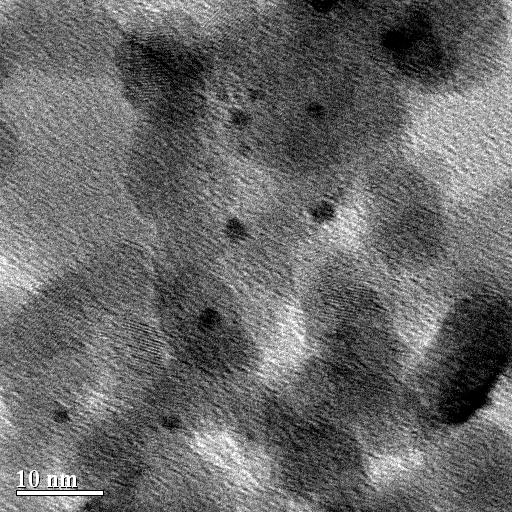


**Figure S13.** HRTEM image of 1% Au/TiO_2_ catalyst.

**Figure S14.** Arrhenius plot for Pt_1_/MoC catalyzed selective hydrogenation of quinoline. Reaction conditions: 0.005 mol% Pt, 2 mL toluene, 4.15 mmol quinoline (0.5 mL), 2 MPa H_2_, 60−100 ^o^C, 1 h. The conversions were kept below 20% for calculation of initial TOF.

**Figure S15.** Recycling of Pt_1_/MoC catalyst for selective hydrogenation of quinoline. Reaction conditions for each run: 0.01 mol% Pt, 2 mL toluene, 4.15 mmol quinoline (0.5 mL), 2 MPa H_2_, 100 ^o^C, 10 h.

**Figure S16.** (a) HAADF-STEM and (b) aberration-corrected STEM-HAADF images of the used Pt_1_/MoC catalyst. Inset in (b) is the FFT data showing the single-crystal structure of the used Pt_1_/MoC. Atomically dispersed Pt atoms in image (b) are highlighted by the red circles.

**Figure S17.** (a) TEM image and (b) HAADF-STEM-EDS elemental mappings of Mo, C, and Pt of the used Pt_1_/MoC catalyst.

**Figure S18.** Aberration-corrected STEM-HAADF image of the used Pt_1_/MoC catalyst. Atomically dispersed Pt atoms are highlighted by the red circles.

**Table S4.** Kinetic isotope effects of selective hydrogenation of quinoline using Pt_1_/MoC.^a^

| Entry | Reactants | TOF (h^−1^) | KIE^b^ |
| --- | --- | --- | --- |
| 1 | Quinoline + H_2_ | 3672 | — |
| 2 | Quinoline + HD | 1021 | 3.6 |
| 3 | Quinoline + D_2_ | 725 | 5.1 |

^a^ Reaction conditions: 0.005 mol% Pt, 2 mL toluene, 4.15 mmol quinoline, 2 MPa H_2_ or HD or D_2_, 100 ^o^C, 1 h. The conversions were kept below 20% for calculation of initial TOF.

^b^ KIE = TOF (Entry 1)/TOF (Entry n) (n = 2 or 3).

**Table S5.** Study of various solid catalysts for the selective hydrogenation of crotonaldehyde.^a^

| Entry | Catalyst | Conv. (%) | Sel. to B (%) | Sel. to C (%) | Sel. to D (%) | TOF (h^−1^)^b^ |
| --- | --- | --- | --- | --- | --- | --- |
| 1 | Pt_1_/MoC | 86.2 | 53.4 | 32.1 | 14.5 | 466 |
| 2^c^ | Pt_1_/MoC | 72.5 | 41.6 | 31.3 | 27.1 | 1216 |
| 3^d^ | 0.2% Pt/MoC | 60.5 | 35.4 | 31.5 | 33.1 | 429 |
| 4^d^ | 0.5% Pt/MoC | 51.7 | 36.8 | 35.3 | 27.9 | 378 |
| 5 | 1% Pt/MoC | 84.3 | 35.6 | 30.3 | 34.1 | 299 |
| 6 | 2% Pt/MoC | 70.9 | 35.2 | 27.6 | 37.2 | 248 |

^a^ Reaction conditions: 1 μmol Pt, 6.07 mmol crotonaldehyde (0.5 mL), 2 mL ethanol, 2 MPa H_2_, 100 ^o^C, 6 h.

^b^ Average TOF based on total Pt sites and the conversion of crotonaldehyde. ^c^ 0.25 μmol Pt. ^d^ 0.5 μmol Pt.

**Supplementary references**

[S1] L. Lin, W. Zhou, R. Gao, S. Yao, X. Zhang, W. Xu, S. Zheng, Z. Jiang, Q. Yu, Y. W. Li, C. Shi, X. D. Wen, D. Ma, *Nature* **2017**, *544*, 80−83.

[S2] Q. Y. Bi, X. L. Du, Y. M. Liu, Y. Cao, H. Y. He, K. N. Fan, *J. Am. Chem. Soc.* **2012**, *134*, 8926−8933.

[S3] M. Yang, S. Li, Y. Wang, J. A. Herron, Y. Xu, L. F. Allard, S. Lee, J. Huang, M. Mavrikakis, M. Flytzani-Stephanopoulos, *Science* **2014**, *346*, 1498–1501.

[S4] Y. Chen, S. Ji, W. Sun, W. Chen, J. Dong, J. Wen, J. Zhang, Z. Li, L. Zheng, C. Chen, Q. Peng, D. Wang, Y. Li, *J. Am. Chem. Soc.* **2018**, *140*, 7407−7410.

[S5] [H](https://www.nature.com/articles/s41565-018-0089-z#auth-1). Li, [L](https://www.nature.com/articles/s41565-018-0089-z#auth-2). Wang, [Y](https://www.nature.com/articles/s41565-018-0089-z#auth-3). Dai, [Z](https://www.nature.com/articles/s41565-018-0089-z#auth-4). Pu, [Z](https://www.nature.com/articles/s41565-018-0089-z#auth-5). Lao, [Y](https://www.nature.com/articles/s41565-018-0089-z#auth-6). Chen, [M](https://www.nature.com/articles/s41565-018-0089-z#auth-7). Wang, [X](https://www.nature.com/articles/s41565-018-0089-z#auth-8). Zheng, [J](https://www.nature.com/articles/s41565-018-0089-z#auth-9). Zhu, [W](https://www.nature.com/articles/s41565-018-0089-z#auth-10). Zhang, [R](https://www.nature.com/articles/s41565-018-0089-z#auth-11). Si, [C](https://www.nature.com/articles/s41565-018-0089-z#auth-12). Ma,  [J](https://www.nature.com/articles/s41565-018-0089-z#auth-13). Zeng, *Nat. Nanotechnol.* **2018**, *13*, 411–417.

[S6] [Y. Kim](https://onlinelibrary.wiley.com/action/doSearch?ContribAuthorStored=Kim%2C+Yong-Tae), [K. Ohshima](https://onlinelibrary.wiley.com/action/doSearch?ContribAuthorStored=Ohshima%2C+Kazuyoshi), [K. Higashimine](https://onlinelibrary.wiley.com/action/doSearch?ContribAuthorStored=Higashimine%2C+Koichi), [T. Uruga,](https://onlinelibrary.wiley.com/action/doSearch?ContribAuthorStored=Uruga%2C+Tomoya) [M. Takata,](https://onlinelibrary.wiley.com/action/doSearch?ContribAuthorStored=Takata%2C+Masaki) [H. Suematsu,](https://onlinelibrary.wiley.com/action/doSearch?ContribAuthorStored=Suematsu%2C+Hiroyoshi) [T. Mitani](https://onlinelibrary.wiley.com/action/doSearch?ContribAuthorStored=Mitani%2C+Tadaoki), *Angew. Chem. Int. Ed.* **2006**, *45*, 407–411.

[S7] H. Yan, H. Cheng, H. Yi, Y. Lin, T. Yao, C. Wang, J. Li, S. Wei, J. Lu, *J. Am. Chem. Soc.* **2015**, *137*, 10484–10487.

[S8] [S](https://www.nature.com/articles/s41565-018-0197-9#auth-1). Wei, [A](https://www.nature.com/articles/s41565-018-0197-9#auth-2). Li, [J](https://www.nature.com/articles/s41565-018-0197-9#auth-3). Liu, [Z](https://www.nature.com/articles/s41565-018-0197-9#auth-4). Li, [W](https://www.nature.com/articles/s41565-018-0197-9#auth-5). Chen, [Y](https://www.nature.com/articles/s41565-018-0197-9#auth-6). Gong, [Q](https://www.nature.com/articles/s41565-018-0197-9#auth-7). Zhang, [W](https://www.nature.com/articles/s41565-018-0197-9#auth-8). Cheong, [Y](https://www.nature.com/articles/s41565-018-0197-9#auth-9). Wang, [L](https://www.nature.com/articles/s41565-018-0197-9#auth-10). Zheng, [H](https://www.nature.com/articles/s41565-018-0197-9#auth-11). Xiao, [C](https://www.nature.com/articles/s41565-018-0197-9#auth-12). Chen, [D](https://www.nature.com/articles/s41565-018-0197-9#auth-13). Wang, [Q](https://www.nature.com/articles/s41565-018-0197-9#auth-14). Peng, [L](https://www.nature.com/articles/s41565-018-0197-9#auth-15). Gu, [X](https://www.nature.com/articles/s41565-018-0197-9#auth-16). Han, [J](https://www.nature.com/articles/s41565-018-0197-9#auth-17). Li, Y. Li, *Nat. Nanotechnol.* **2018**, *13*, 856–861.

[S9] Y. Yao, Z. Huang, P. Xie, L. Wu, L. Ma, T. Li, Z. Pang, M. Jiao, Z. Liang, J. Gao, Y. He, D. J. Kline, M. R. Zachariah, C. Wang, J. Lu, T. Wu, T. Li, C. Wang, R. Shahbazian-Yassar, L. Hu, *Nat. Nanotechnol.* **2019**, *14*, 851–857.

[S10] Y. P. Sun, H. Y. Fu, D. L. Zhang, R. X. Li, H. Chen, X. J. Li, *Catal. Commun.* **2010**, *12*, 188−192.

[S11] N. Hashimoto, Y. Takahashi, T. Hara, S. Shimazu, T. Mitsudome, T. Mizugaki, K. Jitsukawa, K. Kaneda, *Chem. Lett.* **2010**, *39*, 832−834.

[S12] H. Mao, C. Chen, X. Liao, B. Shi, *J. Mol. Catal. A* **2011**, *341*, 51−56.

[S13] D. Ren, L. He, L. Yu, R. S. Ding, Y. M. Liu, Y. Cao, H. Y. He, K. N. Fan, *J. Am. Chem. Soc.* **2012**, *134*, 17592−17598.

[S14] Y. Gong, P. Zhang, X. Xu, Y. Li, H. Li, Y. Wang, *J. Catal.* **2013**, *297*, 272−280.

[S15] H. Mao, J. Ma, Y. Liao, S. Zhao, X. Liao, *Catal. Sci. Technol.* **2013**, *3*, 1612−1617.

[S16] M. Yan, T. Jin, Q. Chen, H. E. Ho, T. Fujita, L. Y. Chen, M. Bao, M. W. Chen, N. Asao, Y. Yamamoto, *Org. Lett.* **2013**, *15*, 1484−1487.

[S17] L. Tao, Q. Zhang, S. S. Li, X. Liu, Y. M. Liu, Y. Cao, *Adv. Synth. Catal.* **2015**, *357*, 753−760.

[S18] M. Tang, J. Deng, M. Li, X. Li, H. Li, Z. Chen, Y. Wang, *Green Chem.* **2016**, *18*, 6082−6090.

[S19] L. Bai, X. Wang, Q. Chen, Y. Ye, H. Zheng, J. Guo, Y. Yin, C. Gao, *Angew. Chem. Int. Ed.* **2016**, *55*, 15656−15661.

[S20] X. Wang, W. Chen, L. Zhang, T. Yao, W. Liu, Y. Lin, H. Ju, J. Dong, L. Zheng, W. Yan, X. Zheng, Z. Li, X. Wang, J. Yang, D. He, Y. Wang, Z. Deng, Y. Wu, Y. Li, *J. Am. Chem. Soc.* **2017**, *139*, 9419−9422.
